# Pre-Attentive, Context-Specific Representation of Fear Memory in the Auditory Cortex of Rat

**DOI:** 10.1371/journal.pone.0063655

**Published:** 2013-05-06

**Authors:** Akihiro Funamizu, Ryohei Kanzaki, Hirokazu Takahashi

**Affiliations:** 1 JSPS Research fellow, Chiyoda-ku, Tokyo, Japan; 2 Neural Computation Unit, Okinawa Institute of Science and Technology, Kunigami, Okinawa, Japan; 3 Graduate School of Information Science and Technology, The University of Tokyo, Bunkyo-ku, Tokyo, Japan; 4 Research Center for Advanced Science and Technology, The University of Tokyo, Meguro-ku, Tokyo, Japan; 5 PRESTO, JST, Kawaguchi, Saitama, Japan; Tokai University, Japan

## Abstract

Neural representation in the auditory cortex is rapidly modulated by both top-down attention and bottom-up stimulus properties, in order to improve perception in a given context. Learning-induced, pre-attentive, map plasticity has been also studied in the anesthetized cortex; however, little attention has been paid to rapid, context-dependent modulation. We hypothesize that context-specific learning leads to pre-attentively modulated, multiplex representation in the auditory cortex. Here, we investigate map plasticity in the auditory cortices of anesthetized rats conditioned in a context-dependent manner, such that a conditioned stimulus (CS) of a 20-kHz tone and an unconditioned stimulus (US) of a mild electrical shock were associated only under a noisy auditory context, but not in silence. After the conditioning, although no distinct plasticity was found in the tonotopic map, tone-evoked responses were more noise-resistive than pre-conditioning. Yet, the conditioned group showed a reduced spread of activation to each tone with noise, but not with silence, associated with a sharpening of frequency tuning. The encoding accuracy index of neurons showed that conditioning deteriorated the accuracy of tone-frequency representations in noisy condition at off-CS regions, but not at CS regions, suggesting that arbitrary tones around the frequency of the CS were more likely perceived as the CS in a specific context, where CS was associated with US. These results together demonstrate that learning-induced plasticity in the auditory cortex occurs in a context-dependent manner.

## Introduction

State-dependent neural representation is found in both the sensory (e.g., [1]–[3]) and motor systems [4], suggesting that neurons multiplex their function to perform different analyses according to context. In the auditory cortex, top-down attention is a predominant mechanism inducing the rapid, adaptive plasticity that reshapes receptive fields in a context-dependent manner [5]–[7]. Pre-attentively (under anesthesia), the receptive fields are also rapidly modulated by bottom-up stimulus properties [8] such as stimulus density [9], bandwidth [10], envelope [11], and context [12]. Such context-dependent modulation of receptive fields is critically involved for contrast gain control [13], possibly through synaptic depression [14], [15] and/or other network mechanisms [11], [16]. Additionally, past experiences such as passive sound exposure and active learning influence the pre-attentive cortical representation (for reviews: [17]–[19]). However, little attention has been paid to the rapid, context-dependent modulation of such plasticity.

The auditory cortex is critical for storage of emotional meaning of sounds [20], [21] and in foreground-background decomposition of sound information [22]–[25]. In the real world, both meaningful foreground sounds and meaningless background contexts are subject to change; thus, the optimal receptive field should be determined not only by bottom-up stimulus properties, but also by meaning of sound within a given context. We hypothesize here that context-specific learning leads to pre-attentively modulated, multiplex representation in the auditory cortex. Such representation would be beneficial to organisms because multiplex representation enriches the functional diversity of each neuron and neural circuit without allocating additional resources of attention.

Context-dependent fear extinction studies have demonstrated that contextual modulation of activities in the lateral amygdala is a putative mechanism for the context-specific expression of fear memory [26]. Accumulating evidence shows that such modulation is enabled by varied brain regions, including hippocampus, striatum, thalamus, prefrontal cortex, and limbic system [27]–[32]. However, little attention has been paid to the sensory cortex.

In the present study, we test whether, and how the auditory cortex represents context-specific fear memory. The context is provided by auditory, non-spatial cues, to avoid a major contribution of hippocampus to the task [28], [33], [34]. We investigate cortical map plasticity in anesthetized rats, which have been conditioned in a context-dependent manner, such that conditioned stimulus (CS, tone) and unconditioned stimulus (US, foot-shock) are associated only under noisy auditory context but not in silence. Our results suggest that cortical plasticity of pre-attentive modulation is effective, such that arbitrary tones around the CS are more likely perceived as the CS in a specific context, where CS is associated with US.

## Materials and Methods

This study was carried out in strict accordance with “Guiding Principles for the Care and Use of Animals in the Field of Physiological Science” by the Japanese Physiological Society. The protocol was approved by the Committee on the Ethics of Animal Experiments at Research Center for Advanced Science and Technology, The University of Tokyo (Permit Number: RAC07110). All surgery was performed under isoflurane anesthesia, and all efforts were made to minimize suffering. Both behavioral and electrophysiological experiments were performed in a sound attenuating chamber.

### Subjects

Twenty six male Wistar rats, at postnatal week 9 or 10, with a body weight of 250 to 350 g, were used in this study. Ten rats were assigned to the conditioned group, where context-dependent auditory fear conditioning was conducted, and the remaining 16 rats were assigned to the naïve group, which received no training and served as home-cage control. Of 16 naïve rats, 10 rats were used in electrophysiological recording, and 6 rats were used to assess naïve behaviors in response to auditory stimuli.

### Context-dependent auditory fear conditioning

Behavioral experiments were performed in a custom-made experimental chamber (O'hara & Co. Ltd., Tokyo, Japan) measuring 24×24×35 cm. One day prior to the conditioning, rats were placed in the experimental chamber, and pre-exposed to a pure tone with a frequency of 20 kHz and an intensity of 70 dB SPL (sound pressure level in decibels with respect to 20 μPa), and to a white noise (50 dB SPL). Each stimulus was 20-s duration, and was presented 10 times in a pseudo-random order, with a pseudo-random inter-stimulus interval ranging from 30 to 60 s. The total time of the pre-exposure was approximately 15 min. The acoustic stimuli were delivered from a speaker placed at the ceiling of experimental chamber, and the speaker was used in all the following behavioral experiments. Prior to the experiments, acoustic calibration was performed with a 1/4-inch microphone (Brüel and Kjaer, 4939). The experimental chamber had 2 black and 2 transparent acrylic walls and a black-metallic grid floor. The light in the chamber was turned on during both the pre-exposure and the conditioning.

**Figure 1:**
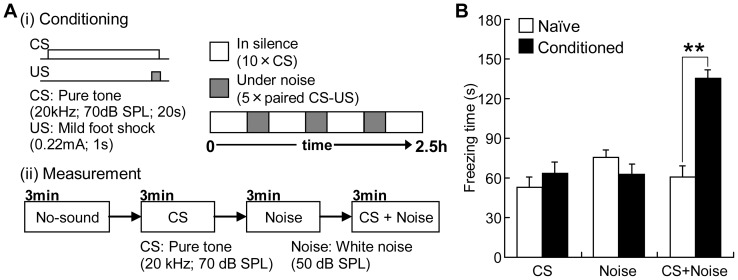
Context-dependent auditory fear conditioning. (A) Procedure used for conditioning (i) and measurement (ii). (i) In the conditioning session, 4 silent and 3 noise conditions were prepared, and were sequentially alternated. In a silent-condition block, only a conditioned stimulus (CS) consisting of a 20-kHz tone was presented 10 times. In a noise-condition block, a white noise stimulus was continuously presented during the block, and an unconditioned stimulus (US) consisting of an electrical foot shock was associated with the CS 5 times. The total time of the conditioning session was approximately 2.5 h. (ii) The measurement of freezing time of rats was conducted the day after the conditioning session. CS only, noise only, and CS under noise were presented in order each for 3 min. (B) Freezing times of rats. Asterisks indicate the significance of post-hoc analyses (Mann-Whitney U-test: **, p<0.01).

Figure 1A (i) shows the procedure used for context-dependent auditory fear conditioning. The conditioning was conducted in the experimental chamber, which was identical to the one used in the pre-exposure session. The conditioning consisted of 4 silent condition blocks, and 3 noise condition blocks, which were alternated sequentially. In a silent-condition block, a CS of pure tone (20 kHz, 70 dB SPL, 20 s) was presented 10 times, with a pseudo-random inter-stimulus interval ranging from 1 to 4 min. In a noise-condition block, a continuous white noise stimulus (50 dB SPL) was presented throughout the block, and the last 1 s of the CS was associated with an US consisting of an electrical foot shock (0.22 mA, 1 s) delivered through the metal-grid floor. These CS-US pairs were presented 5 times in each block. The inter-stimulus interval between CS-US pair was pseudo random, ranging from 1 to 4 min. The total time of the conditioning session was approximately 2.5 h.

The day after the conditioning (i.e., approximately 24 h after), a measurement session was conducted, as shown in Fig. 1A (ii), where the freezing times of rats were measured with presentation of the CS only, white noise only, and CS with white noise. Each stimulus was presented for 3 min, and the inter-stimulus interval was determined, such that the rats moved for at least 1 min during the interval. In order to differentiate the contexts of conditioning from those of the measurement session and to prevent a contextual fear, the chamber used for the behavioral measurements had 4 black walls and a paper towel on the floor, and the light of chamber was turned off [28], [35].

Freezing times were measured using images recorded by a camera placed above the measurement chamber. Images were captured every 0.5 s, and binarized in order to quantify the movement of rats. Freezing time was defined as an accumulating time period, during which image-by-image differences of white areas (i.e., the rat) did not reach an empirical threshold. The threshold was determined such that image-based and human-observation-based freezing times showed a concordance of 90% or more [36].

### Electrophysiological mapping

The day after the freezing measurements were made (i.e., approximately 48 h after the conditioning sessions), tone-evoked neural activities were recorded in the auditory cortex both under silent and noise conditions. Under the noise condition, white noise (40 dB SPL) was continuously presented throughout the recording session, while the silent condition had no acoustic background.

Rats were anesthetized with isoflurane (3% at induction and 1–2% for maintenance), and were fixed using a custom-made head-holding device. Atropine sulfate (0.1 mg/kg) was administered at the beginning of the surgery and every 8 h thereafter to reduce the viscosity of bronchial secretions. A heating blanket was used to maintain body temperature at around 37°C. The temporal muscle, cranium, and dura overlying the auditory cortex were surgically removed, and the exposed cortical surface was covered with silicone oil in order to prevent desiccation. Cisternal cerebrospinal fluid drainage was performed to minimize cerebral edema. Respiratory rate, heart rate and hind-paw withdrawal reflexes were monitored to maintain an adequate anesthetic level as uniformly as possible throughout the recording procedure.

Acoustic stimuli were given as tone bursts with a 5-ms plateau, and 5-ms rise/fall times. The test tones had frequencies ranging from 1 to 50 kHz, with 1/3-octave increments (1, 1.3, 1.6, 2.0,…, 32, 40, 50), and intensities from 30 to 70 dB SPL, with 5-dB increments. With the 18 test frequencies and 9 intensities, 162 test tones were used in total. Each tone was presented 20 times, in a pseudo-random order. These stimuli were delivered to the left (contralateral) pinna, every 200 ms, through the sound delivery tube of an electrostatic speaker (Tucker-Davis Technologies, Inc., EC1), which was calibrated with a 1/4-inch microphone (Brüel and Kjaer, 4939).

Multiunit activities were recorded using teflon-coated tungsten microelectrodes (California fine wire Co.) [37]. Each insulated probe had a diameter of 50 μm in total, with a bare metal diameter of 30 μm (∼100 kΩ impedance at 1 kHz). An array of 14 or 18 electrodes, aligned in a row at 350-μm intervals, were inserted vertically to the pial surface, and advanced toward a depth of 400–600 μm. Neural signals were recorded with an amplification gain of 1000, digital filter bandpass of 0.75–7.5 kHz, and sampling frequency of 30 kHz (Cyberkinetics Inc.; Cerebus Data Acquisition System). The electrode array was inserted repeatedly (about 17 times per subject) from ventral to dorsal areas, in order to map the entire cortical region of interest.

### Data analysis

All analyses and statistical tests were performed offline with custom-written Matlab (The Mathworks, Natick, MA) and R (http://www.r-project.org/) programs.

#### Characterization of neural activities

The frequency response area (FRA) at each recording site was determined under both silent and noise conditions, on the basis of multiunit activities between 5 and 50 ms from stimulus onset, in response to the 18 test frequencies at 9 intensities. In accordance with previous studies (e.g., [38]–[40]), the evoked response to each tone was identified when the average spike rate within the time-window was larger than the mean plus 1.64 standard deviations, i.e., 90% confidence interval, of a mean spontaneous rate. The mean spontaneous rate was defined as the firing rate during the first 3 ms after stimulus onset, averaged across all stimuli [39], because no auditory-evoked activities were observed at the post-stimulus latency of 5 ms or earlier [40]–[42]. At each test intensity, the best frequency (BF) of each recording site was determined, defined as the frequency at which a test tone evoked the largest response. A characteristic frequency (CF) was approximately determined, at which test tones evoked a response at the lowest intensity, or the largest response at 30 dB SPL, the minimum intensity used in this experiment [43]. This approximated CF was used because precise calibration of test intensity was not possible below 30 dB SPL in our system. The bandwidth of FRA was determined for each test intensity. The latency of tone-evoked responses was defined as the time when the maximum number of spikes was recorded from stimuli onsets, on the basis of a post-stimulus time histogram (PSTH) with a bin width of 1 ms.

In accordance with previous studies [41], [42], the borders of auditory fields were determined by the discontinuity of CF, the bandwidths, and the latency gradients in the silent condition. A1 was defined based on the short peak latency in the dorsal auditory field containing a high-to-low tonotopic gradient, running along the rostral-to-caudal axis. A tonotopic reversal at the anterior periphery of A1 was defined as the border between A1 and the anterior auditory field (AAF), which also had the short peak latency, and a high-to-low tonotopic gradient along the posterodorsal-to-anteroventral axis. Tone response areas that abutted a ventral border of A1, and a posterior border of AAF, were the ventral and suprarhinal auditory fields (VAF and SRAF), which had longer latency responses than A1 and AAF, with clear tonotopic gradients. The posterior auditory field (PAF) was defined posteriorly to A1, with tonotopic discontinuity and a longer latency. Inconsistent with previous studies [41], [44], [45], the tonotopic gradient in PAF was not always clear, possibly due to our sparser measurements. The anterior ventral auditory field (AVAF) was defined based on tonotopic discontinuity at a ventral border of AAF and anteroventral border of SRAF [46]. The ventral part of AVAF could not be fully characterized because of the rhinal vein [47]. Other small areas were also found in the anterior or posteroventral part of auditory cortical fields, possibly corresponding to the insular cortex [47], or to another undefined field (e.g., [48]). Fully characterized areas, i.e., A1, AAF, VAF, and SRAF, were our focus of interest in the analyses.

To visualize the topography of the auditory cortex, the Voronoi tessellation procedure was used to create tessellated polygons, with their centers corresponding to recording sites [49], [50]. The CFs were then illustrated by color-coded polygons. These polygons were used to calculate the tone response areas of auditory cortical fields.

#### Cortical recruitment functions

The cortical recruitment functions (CRFs) were calculated as population characteristics under both silent and noise conditions. CRF measured the percentage of recording sites activated by each test tone of a specific frequency and intensity [41], [42], [49]. The presence of activation at each recording site to a given tone was determined in accordance with FRA.

#### Accuracy of frequency representations

To quantify how accurately neurons represented a test frequency of tone, we defined and measured an index of probabilistic encoding specificity, termed “an encoding accuracy index.” Considering that, when a test frequency was *f_presented_* and a post-stimulus spike count of a given neuron was *x*, this neuron predicted *f_presented_* as *f_estimate_*, the probability (i.e., accuracy) to predict *f_presented_* as *f_estimate_*, i.e., *p(f_estimate_* | *f_presented_)*, was described as follows [51], [52]:
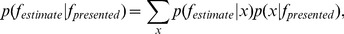
(1)where *p(f_estimate_* | *x)* was the probability to estimate a test frequency from a given spike count (i.e., decoding) and *p(x* | *f_presented_)* was the probability to obtain a spike count in response to a given test tone (i.e., encoding). Therefore, Eq. 1 describes how the accuracy of test frequency was deteriorated by the encoding and decoding. Both *p(f_estimate_* | *x)* and *p(x* | *f_presented_)* were obtained with a table of a set of tone-evoked spike counts between 5 and 50 ms from the stimulus onset, X (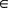
0, 1, 2,…) and a set of test frequencies, F (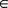
1, 1.3, 1.6,…, 32, 40, 50). This encoding accuracy index quantifies the accuracy of frequency representation; for example, when a neuron is active only for a specific test frequency, the accuracy becomes high at the corresponding frequency.

## Results

### Behavior

Behaviors were first characterized in Fig. 1B to verify context-dependent auditory fear learning. In the conditioned group, the CS (20-kHz) tone presented under the noise condition led to significantly longer freezing times (136±6.50 s (mean ± standard error, here and hereafter)) than either the CS tone present in a silence context (63.3±8.54 s), or a noise background without the CS (62.7±7.79 s) (two-way ANOVA with Mendoza's multisample sphericity test (λ(5)  = 0.0355, p = 0.327): F(2,28)  = 15.4, (F-value with 2 and 28 degrees of freedom in between- and within-groups, respectively, was 15.4), p = 3.09E-5; Post-hoc Wilcoxon signed rank test: CS + Noise vs. CS, signed rank  = 0, p = 0.00195; CS + Noise vs. Noise, signed rank  = 0, p = 0.00195). In contrast, the CS tone presented in silence, and noise without the CS did not lead to significantly different freezing times (Post-hoc test: signed rank  = 26, p = 0.922). In the naïve group, on the other hand, the freezing times under the above three conditions did not show significant differences (Post-hoc test: signed rank  = 6–2, p = 0.438–0.0938). When compared between the naïve and conditioned groups, the freezing times during the CS under noise were significantly longer in the conditioned group than in the naïve group (two-way ANOVA: F(1,14)  = 9.91, p = 0.00713; Post-hoc Mann-Whitney U-test: rank sum  = 21, p = 2.50E–4), while the freezing times in other 2 conditions had no significant differences (Post-hoc test: rank sum  = 43 and 58, p = 0.428 and 0.474). Thus, the freezing times during the CS under noise in the conditioned group were selectively long (interaction term in two-way ANOVA: F(2,28)  = 18.5, p = 7.52E–6). These results together indicate that the present task successfully conditioned rats in a context-dependent manner.

### Cortical mapping

**Figure 2:**
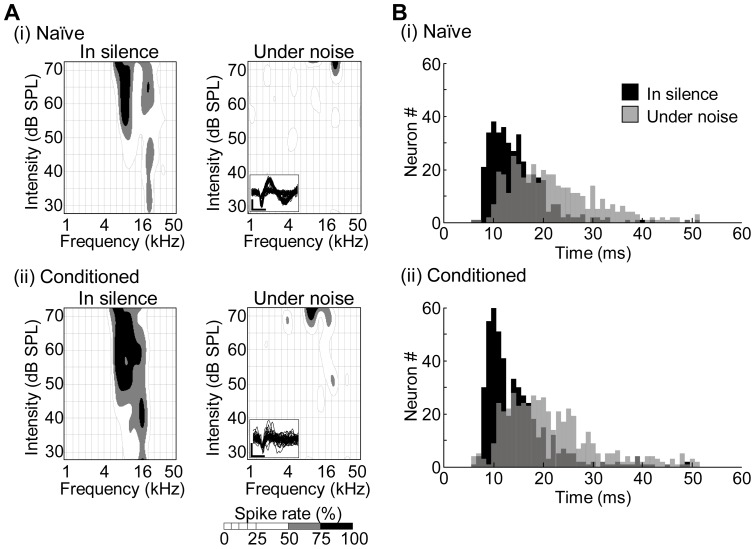
Characterization of multi-unit activities. (A) Representative frequency response area (FRA) in the auditory cortex in a naïve (i) and a conditioned rat (ii). The FRAs were different under silent and noise conditions. Spike rates are shown in gray scale for a given pair of test frequencies (abscissa) and intensities (ordinate). The insets at lower left show action potential waveforms. Scale bar: vertical axis, 50 μV; horizontal axis, 0.5 ms. (B) Histogram of PSTH peak latency in silence (black) and under the noise condition (gray).

In electrophysiological experiments, we first investigated how the conditioning modified the tonotopic map in the auditory cortex because the map plasticity is a major hallmark of learning [17]–[19]; of our particular interest here is context-dependent modification of tonotopic map. To reveal learning-induced, context-dependent plasticity, we have to characterize an interaction of learning effects (i.e., naïve vs. conditioned groups) and context effects (i.e., silent vs. noise conditions), in addition to both of these individual main effects. Figures 2A (i) and (ii) show representative tone-evoked multi-unit activities recorded from both a naïve and a conditioned rat, respectively, showing that FRA properties such as CF and BF at a given intensity in silence and noise, were not identical. For example, neurons in Fig. 2A (i) had a clear CF at 20 kHz in silence, but did not show clear tone-evoked activities under the noise condition. In Fig. 2A (ii), when the background auditory context changed from silence to noise, CFs shifted from 16 kHz to 20 kHz, and BFs at 70 dB SPL from 6.4 kHz to 10 kHz. Thus, CFs and BFs were dependent on the auditory context. Figures 2B (i) and (ii) summarize PSTH peak latencies of all test trials in the naïve and conditioned groups, respectively. The peak latencies in both groups were comparable (two-way ANOVA: main effect, F(1,1736)  = 0.455, p = 0.500; interaction term, F(1,1736)  = 0.241, p = 0.624), and were shorter in the silent than in the noise condition by approximately 7 ms (two-way ANOVA, F(1,1736)  = 331, p = 7.47E–68) (13.8±0.311 ms vs. 20.9±0.445 ms in the naïve group (Post-hoc Mann-Whitney U-test: Z = 13.0, p = 1.02E–38); 13.7±0.318 ms vs. 20.5±0.421 ms in the conditioned group (Z = 13.9, p = 1.05E–43)).

**Figure 3:**
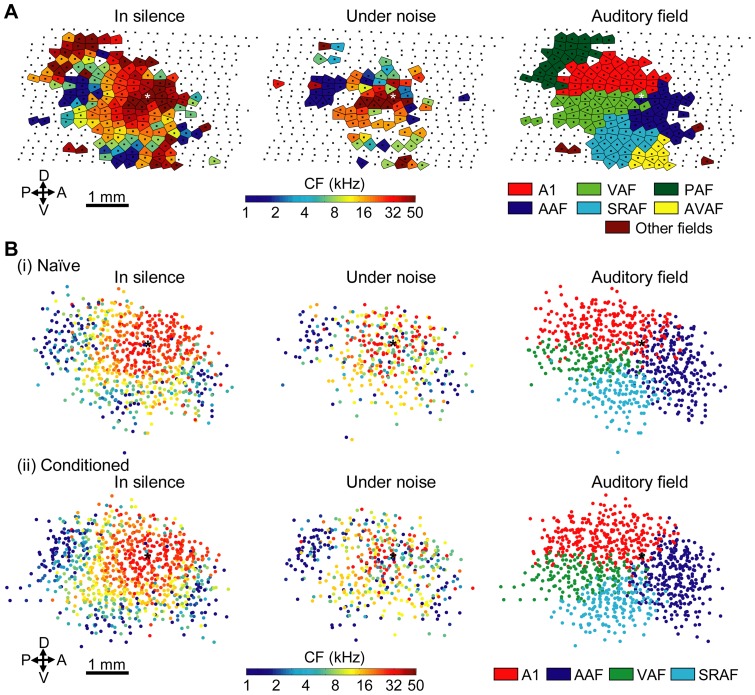
Functional map in the auditory cortex. (A) Representative data from a conditioned rat. Characteristic frequency (CF) under the silent condition (left column) and the noise condition (middle column), and auditory fields (right column) are shown. All the recording sites are indicated by a small ‘x’ (black). The highest CF location was estimated at the ‘*’ mark (white) as the center of the auditory cortex. Areas of the 20-kHz CF (i.e., the CS frequency) are colored in orange. Abbreviations: A, anterior; P, posterior; D, dorsal; V, ventral. (B) Pooled data from the naïve (i) and conditioned groups (ii). Cortical maps from individual subjects were superimposed with the positional reference of the highest CF location, marked with ‘*’ (black), and aligned in the flat-skull plane. Each color dot shows a recording site placed with respect to the position reference. Maps of CFs under the silent condition (left column) and the noise condition (middle column), and auditory fields (right column) are shown. Data from A1, AAF, VAF and SRAF are shown.

Figure 3A shows representative maps of the auditory cortex. When CFs at all of the recording sites were determined in silence (left column), a high CF was generally observed at the center of the auditory cortex, while a low CF was seen at the fringe, under both silent and noise conditions. Yet, this CF gradient became less clear under the noise condition (middle column) than in silence. Based on the tonotopic gradient and response latency in silence, one of the auditory fields, i.e., A1, AAF, VAF, SRAF, PAF and AVAF, was assigned to each recoding site (right column).

In the naïve group (n = 10), we obtained tone response activities in silence from 950 recording sites in multiple auditory fields (A1, 274; AAF, 253; VAF, 116; SRAF, 140; PAF, 40; AVAF, 99; other fields, 28), and 490 sites under the noise condition (A1, 158; AAF, 152; VAF, 56; SRAF, 40; PAF, 4; AVAF, 63; other fields, 17). In the conditioned group (n = 10), tone response activities were obtained from 1161 sites in silence (A1, 318; AAF, 287; VAF, 163; SRAF, 174; PAF, 68; AVAF, 121; other fields, 30), and 565 sites under the noise condition (A1, 189; AAF, 159; VAF, 70; SRAF, 70; PAF, 16; AVAF, 56; other fields, 5). Neural responses in A1, AAF, VAF and SRAF were subsequently characterized in detail, where we had obtained sufficient numbers of tone responsive recording sites with their complete tonotopic gradients in the silent condition. VAF and SRAF were combined together, in accordance with our previous study [42] because the numbers of the recording sites in VAF and SRAF were almost half, compared to those from A1 and AAF, and because VAF and SRAF were adjacent and displayed similar features in their neural activities [41].

For visualization purposes, tonotopic maps from individual subjects under either the silent or noise conditions were pooled in Figs. 3B with an expedient positional reference, where the highest CF was obtained when applying a Gaussian filter to the CF map in silence with a half band width of 350 μm (i.e., the inter-electrode distance) [42], [53]. This pooled data again demonstrated the clear CF gradients in silence in respective auditory fields, which became less clear under the noise condition.

**Figure 4:**
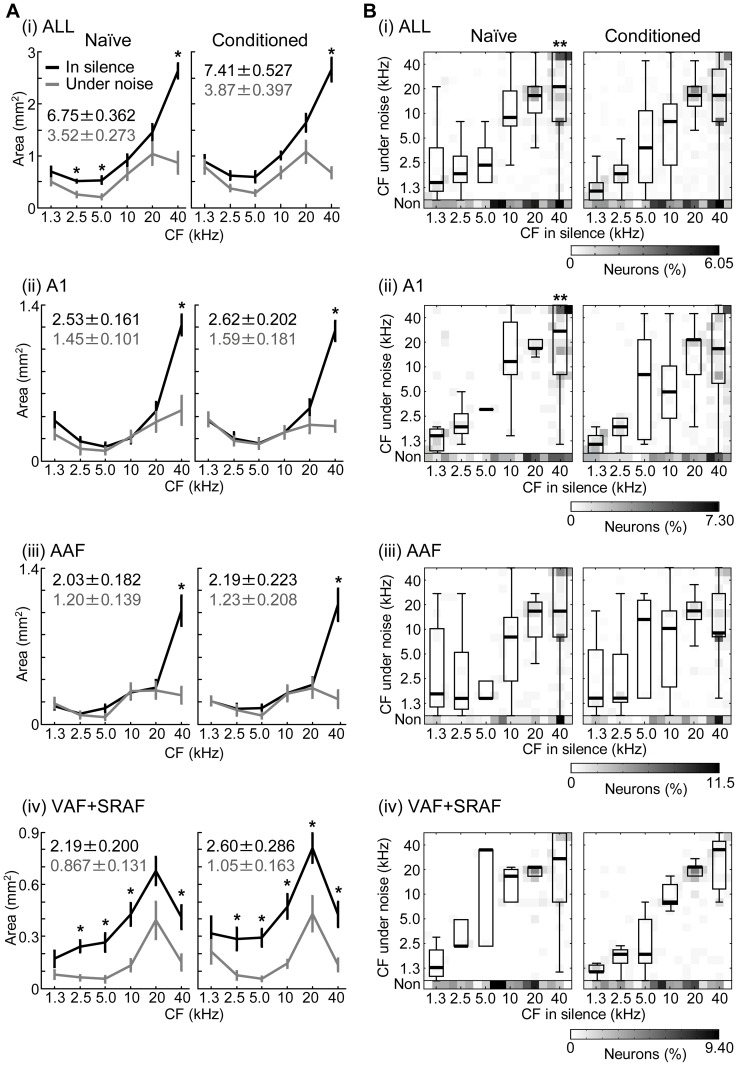
Noise- and conditioning-induced effects on CF. (A) Area breakdown of CF in the whole auditory field (ALL) (i), A1 (ii), AAF (iii) and VAF+SRAF (iv). The absolute areas were measured using Voronoi tessellation polygon maps. Bins were made every 1 octave (i.e., 3 test frequencies). The means and standard errors are shown. Asterisks indicate the significance of post-hoc analyses (Wilcoxon signed rank test after Bonferroni correction for 6 comparisons: *, p<0.05). In each inset, the means and standard errors of the absolute areas that show tone responses are indicated for either silence (black) or noise (gray) condition. (B) CF under silent and noise conditions. Gray scales indicate the number of neurons with a given CF property. ‘Non’ corresponds to loss of tone-evoked responses under the noise condition. Boxplots are overlaid to show the distribution of CF in noise at sites with a given CF in silence. On each box, the central mark is the median, the edges of the box are the 25th and 75th percentiles. The whiskers extend to the most extreme data points not considered outliers, which are larger than the 75th percentiles, or smaller than the 25th percentiles by 1.5 times the inter-quartile range. Asterisks indicate significant differences between pre- and post-conditioning (Mann-Whitney U-test after Bonferroni correction for 6 comparisons: **, p<0.01).

Figure 4A quantitatively compares absolute areas with indicated CF in (i) all fields (i.e., A1, AAF, VAF and SRAF), (ii) A1, (iii) AAF and (iv) VAF + SRAF, based on the Voronoi tessellation procedure. Tone-responsive areas in the silent condition were significantly larger than those under the noise condition in all 4 of the test regions (i–iv) (two-way ANOVA with Mendoza's multisample sphericity test (λ(1)  = 0.0983–0.217, p = 0.7539–0.0893): F(1,18)  = 94.9–195, p = 1.33E–8–4.25E–11; Pos-hoc two-sided paired t-test: naïve, t(9)  = 5.48–8.25, p = 3.89E–4–1.72E–5; conditioned, t(9)  = 7.34–12.4, p = 4.40E–5–6.00E–7), while these areas did not significantly differ between the naïve and conditioned groups under either silent or noise condition (two-way ANOVA: F(1,18)  = 0.141–1.25, p = 0.712–0.278); no significant interaction was found between the naïve-conditioned groups and silent-noise conditions (two-way ANOVA: F(1,18)  = 0.0489–0.943, p = 0.828–0.344). Thus, the tone responsive area globally shrank under the noise condition, yet the conditioning neither affected tone-responsive areas, nor the noise-induced area change.

Under both silent and noise conditions, area breakdowns according to CF did not differ between the naïve and conditioned groups, in all of the auditory fields tested (Figs. 4A (ii–iv)) (two-way ANOVA with Mendoza's multisample sphericity test (λ(1)  = 1.00–0.0419, p = 0.999–0.0144) with Geisser-Greenhouse correction: F(1,18)  = 3.17E–4–4.01, p = 0.999–0.0606); no significant interactions between the naïve-conditioned groups and silent-noise conditions were found (F(1,18)  = 0.00347–1.77, p = 0.954–0.200). In contrast, noise-induced significant area changes were found in some CF regions. In A1 (ii) and AAF (iii), 40-kHz areas significantly decreased under the noise condition in both the naïve and conditioned groups (two-way ANOVA in A1 and AAF: F(1,18)  = 101 and 104, p = 8.56E–9 and 6.41E–9; Post-hoc Wilcoxon signed rank test after Bonferroni correction for 6 comparisons in A1 and AAF: naïve, signed rank  = 1 and 0, p = 0.0234 and 0.0120; conditioned, signed rank  = 0 and 0, p = 0.0120 and 0.0120). In VAF + SRAF (iv), the area reduction under the noise condition was observed in 2.5–40-kHz areas (two-way ANOVA: F(1,18)  = 37.7–52.3, p = 8.51E–6–9.97E–7; Post-hoc test: naïve, signed rank  = 4–0, p = 0.0822–0.0120; conditioned, signed rank  = 1–0, p = 0.0468–0.0120). Thus, the continuous background noise had profound effects on the tonotopic maps in high CF regions in both A1 and AAF (i.e., the core cortex), and entirely in VAF + SRAF (i.e., the belt cortex).

We then investigated how the shrinkage of tone responsive area under noise was associated with shifts of CFs (e.g., Fig. 2A). The noise-induced CF shifts in the respective fields are shown in Figs. 4B (i) – (iv), and quantitatively summarized in Table 1. In the naïve groups, 783 recording sites were tone responsive in silence, while 387 sites were tone responsive in the noise condition; thus, 50.6% (396/783) of the recording sites lost tone-evoked responses under the noise condition. This noise-induced loss of tone-evoked response was significantly larger in VAF + SRAF (the belt cortex) than in A1 + AAF (the core cortex) (168/256 (65.6%) vs. 228/527 (43.3%); z-test: Z = 5.87, p = 2.17E–9). In addition, background continuous noise generally shifted CF toward low frequency; this trend was especially distinct in high CF regions in A1 and AAF, where there was noise induced area reduction in the tonotopic map (Fig. 4A). These trends of noise-induced CF shifts also held true in the conditioned group. In terms of a conditioning effect, noise-induced CF shifts in high CF sites (i.e., 40 kHz) were significantly larger in the conditioned group than in the naïve group across the entire cortex (Mann-Whitney U-test: Z = 3.51, p = 4.41E–4), and in A1 (Z = 3.30, p = 9.71E–4). We then tested the possibility that the conditioning makes tone-evoked responses more robust under the noise condition; this was shown to be true in VAF + SRAF, where tone-evoked responses in the noise condition tended to remain more frequently in the conditioned group than in the naïve group (88/256 (34.4%) vs. 139/337 (41.3%); z-test: Z = 1.71, p = 0.044).

**Table 1:** Breakdown of tone-responsive sites.

	ALL		A1		AAF		VAF+SRAF	
	Naïve	Conditioned	Naïve	Conditioned	Naïve	Conditioned	Naïve	Conditioned
Tone responsive sites in silence	783	942	274	318	253	287	256	337
Tone responsive sites under noise	387 (49.4%)	483 (51.3%)	152 (55.5%)	187 (58.8%)	147 (58.1%)	157 (54.7%)	88 (34.4%)	139 (41.3%)
*- CF under noise > CF in silence*	40 (5.11%)	42 (4.46%)	19 (6.93%)	12 (3.77%)	12 (4.74%)	22 (7.67%)	9 (3.52%)	8 (2.37%)
*- CF under noise* ≒ *CF in silence (within 1/3 oct.)*	189 (24.1%)	219 (23.3%)	77 (28.1%)	84 (26.4%)	60 (23.7%)	46 (16.0%)	52 (20.3%)	89 (26.4%)
*- CF under noise < CF in silence*	158 (20.2%)	222 (23.6%)	56 (20.4%)	91 (28.6%)	75 (29.6%)	89 (31.0%)	27 (10.6%)	42 (12.5%)
Loss of tone-evoked response under noise	396 (50.6%)	459 (48.7%)	122 (44.5%)	131 (41.2%)	106 (41.9%)	130 (45.3%)	168 (65.6%)	198 (58.8%)
Gain of tone-responsiveness only under noise	19	5	6	2	5	2	8	1

### Frequency tuning properties

**Figure 5:**
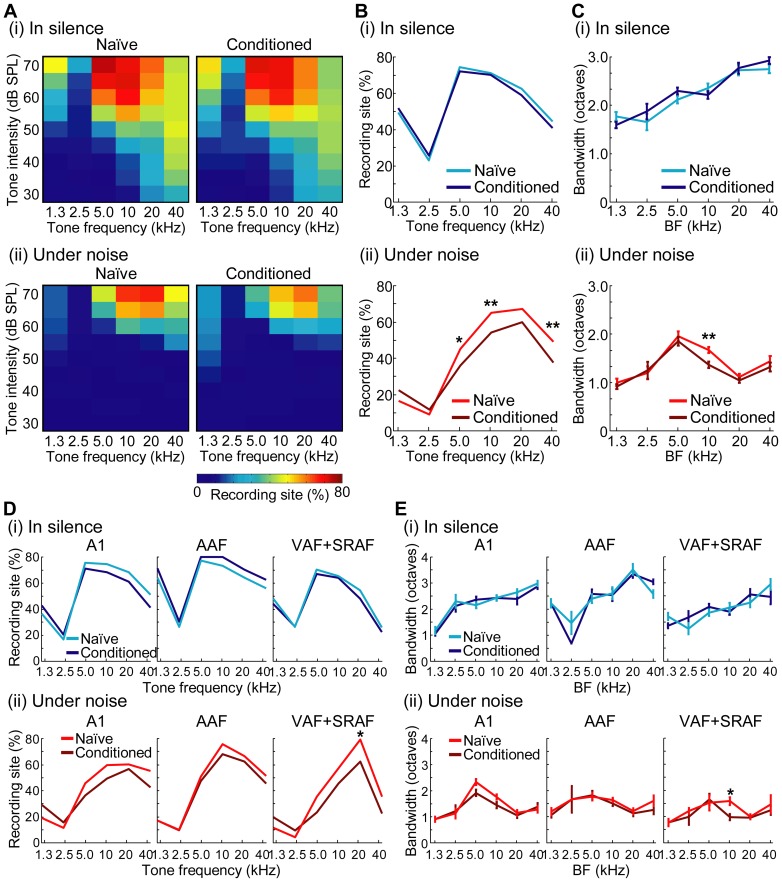
Frequency tuning properties in a population of neural activities. (A) Cortical recruitment functions (CRFs) in silence (i) and noise (ii) in all of the auditory fields. Percentages of recording sites active are given as a function of the test frequency-intensity pair. Test frequencies are combined into 6 groups, each with a 1 octave frequency (i.e., 3 test frequencies). (B) CRFs at 70 dB SPL. Percentages of recording sites active to 70-dB-SPL tones are shown as a function of test frequencies. Asterisks indicate significant differences between the naïve and conditioned groups (z-test after Bonferroni correction for 6 comparisons: *, p<0.05; **, p<0.01). (C) Bandwidths of frequency response areas at 70 dB SPL as a function of the best frequency (BF) of recording site. Asterisks indicate the significance of post-hoc analyses (Mann-Whitney U-test after Bonferroni correction for 6 comparisons: **, p<0.01). (D) CRFs at 70 dB SPL in indicated fields. Asterisks indicate significant differences between the naïve and conditioned groups (Z-test after Bonferroni correction: *, p<0.05). (E) Bandwidths in indicated fields. Asterisks indicate the significance of post-hoc analyses (Mann-Whitney U-test after Bonferroni correction: *, p<0.05).

In addition to CF, we investigated frequency tuning properties in populations of neurons because the CS tone in the fear conditioning was far above the response threshold of neurons. In all of the auditory fields, Fig. 5A investigates frequency tuning properties in silence (i) and noise (ii) in terms of CRF, which is a measure of the spatial extent of activation (i.e., the proportion of responsive sites) to test tones, with an indicated test frequency-intensity pair. CRFs in silence were significantly larger than those in noise in 41 out of 54 test frequency-intensity pairs in the naïve group, and in 46 out of the 54 pairs in the conditioned group (z-test: Z>2.58, p<0.005), indicating that tone-evoked discharges decreased under the noise condition. We then tested whether the conditioning altered CRF properties. At 70 dB SPL, where CS was provided, Figs. 5B (i) and (ii) characterize CRFs in silence and in noise, respectively, demonstrating that the conditioning effects on CRF were found only under the noise condition: CRFs in noise were significantly smaller in the conditioned group than in the naïve group in 3 out of 6 test frequencies (z-test after Bonferroni correction for 6 comparisons: Z = 2.72–3.39, p = 0.0195–0.00209), while CRFs in the silent condition did not show any significant differences between the two groups (Z<1.44, p>0.448). Figure 5D shows the CRF in each field, indicating that reduced activation of CRF was most distinct in VAF + SRAF.

To further test whether such context-dependent conditioning effects of population tuning properties in CRF are associated with those of tuning properties of individual neurons, Fig. 5C shows the bandwidths of FRA at 70 dB SPL, at recording sites with indicated BFs. In terms of context effects, the bandwidths in silence were significantly wider than those under the noise condition in 4 out of 6 BF test regions in the naïve group, and in 5 out of 6 regions in the conditioned group (two-way ANOVA: F(1, 338–564)  = 8.05–386, p = 0.00471–1.85E–61; Post-hoc Mann-Whitney U-test after Bonferroni correction for 6 comparisons: naïve, Z = 4.08–9.31, p = 2.74E–4–7.34E–20; conditioned, Z = 3.20–9.87, p = 0.00840–3.30E–22), indicating that background noise led to a sharpening of the frequency tunings of auditory cortical neurons. In terms of learning effects, the bandwidths in noise at 10-kHz BF sites were significantly narrower in the conditioned than in the naïve group (two-way ANOVA: F(1,564)  = 8.19, p = 0.00436; Post-hoc test: Z = 3.42, p = 0.00372), but this was not true in the silent condition (Z = 1.19, p = 0.2356). Thus, this result suggests that, at the BF region just below the CS frequency, auditory cortical neurons became sharply tuned only under the noise condition after the conditioning. Closer investigation shown in Fig. 5E revealed that this sharpening was significant only in VAF + SRAF (two-way ANOVA: F(1,172)  = 5.43, p = 0.0210; Post-hoc test: Z = 3.03, p = 0.0148). Yet, this learning-induced, context-dependent effect was not very clear according to the significance level of the interaction between the naïve-conditioned groups and silent-noise conditions in all the BF test regions (two-way ANOVA: F(1, 103–564)  = 0.153–2.20, p = 0.697–0.139).

### Accuracy of frequency representations

**Figure 6:**
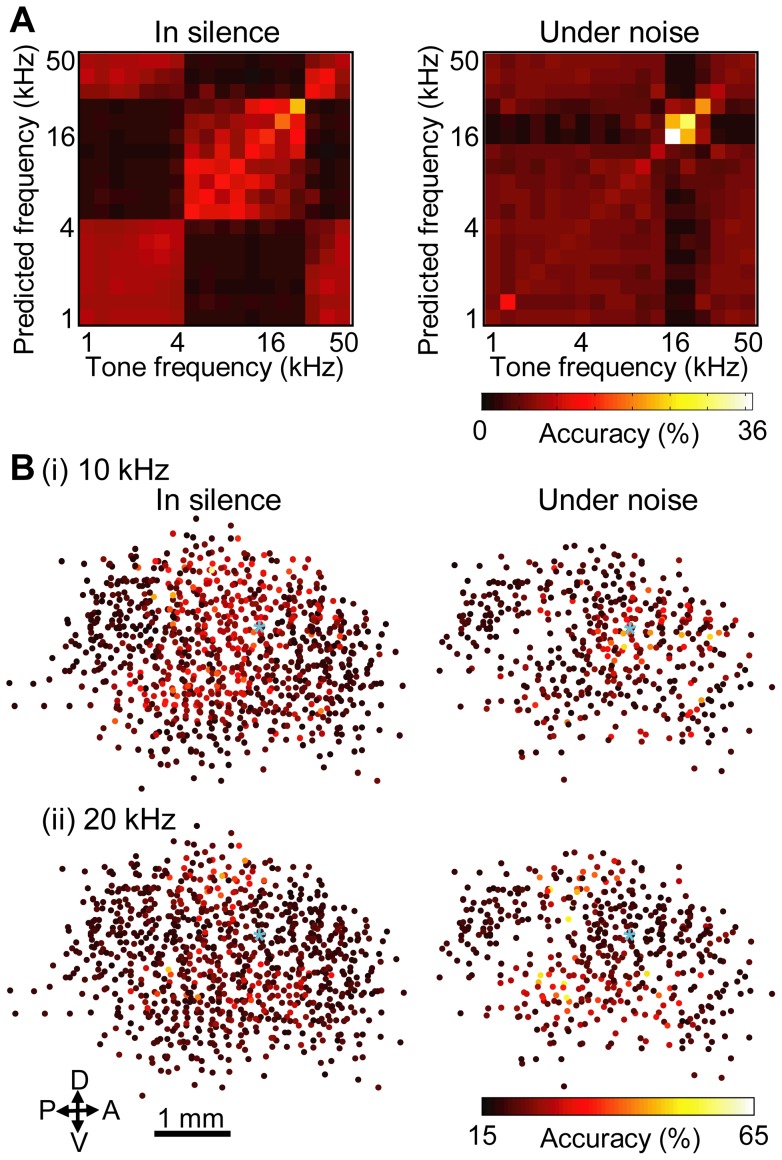
Encoding accuracy. (A) Confusion matrix of encoding accuracy index for a representative recording site. The accuracies of frequency representations are shown for silent and noise conditions. The accuracy is shown in color scale. This neuron coarsely represented 5- to 25-kHz-frequency tones in silence, while 16- and 20-kHz frequencies were represented more accurately under noise. (B) Cortical map of encoding accuracy index from pooled data in the conditioned group: (i) 10 kHz (ii) 20 kHz. The pooled map of CF in the conditioned group is shown for silent and noise conditions (Fig. 3B (ii)). The positional reference of the highest CF location is marked with ‘*’ (light blue). The accuracy of frequency representation for each recording site is shown in color scale. Abbreviations: A, anterior; P, posterior; D, dorsal; V, ventral.

To better interpret how the conditioning-induced plasticity we found impacted tone discrimination or perception, the encoding accuracy index was used to evaluate how accurately each neuron in the auditory cortex represented tone frequencies. Figure 6A shows an example of a confusion matrix of the encoding accuracy index in response to 70-dB-SPL tones. In this representative matrix, the moderate accuracies of 5- to 25-kHz tones in silence imply relatively low resolutions of neural representation of the test stimulus, while the higher accuracies of 16- and 20-kHz tones under the noise condition are indicative of higher resolutions. Measuring the encoding accuracy index at all of the recording sites in the pooled data produced a prediction map of a given test frequency, e.g., 10 kHz and 20 kHz shown in Figs. 6B (i) and (ii), respectively. The accuracy differed between neurons, and was dependent on test frequencies, and the presence of background noise.

**Figure 7:**
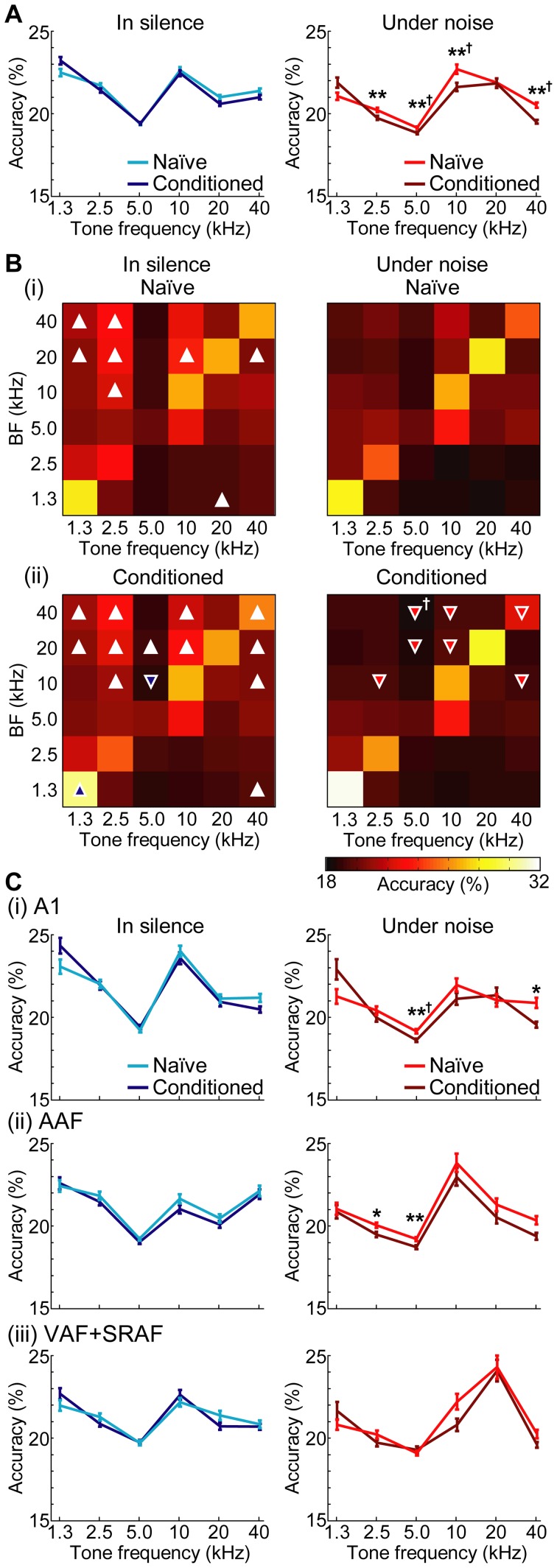
Accuracy of frequency representations at 70 dB SPL. (A) Population average of frequency-representation accuracy as a function of test frequency in silence (left) and noise (right) conditions. The test frequencies are categorized into 6 groups, with a bin width of 1 octave (i.e., 3 test frequencies). Data are presented as means and standard errors. Daggers show significances in the interaction term in two-way ANOVA here and hereafter (†, p<0.05). Asterisks indicate the significance of post-hoc analyses (Mann-Whitney U-test after Bonferroni correction for 6 comparisons, **, p<0.01). (B) Breakdown list of frequency-representation accuracy as a function of the best frequency (BF) of recording sites. The accuracies in (A) are broken down according to the BF of each recording site (ordinate), and shown by color scale. BF and the test frequencies are binned with 1 octave intervals. The diagonal usually had higher accuracies than others, supporting the notion that neurons accurately represented a tone with their own BF. White triangles indicate that the accuracies in silence and noise were significantly different within either the naïve (i) or conditioned group (ii). Blue triangles indicate that accuracies in silence (left) were significantly different between the naïve and conditioned groups, while red triangles indicate significant differences under noise (right). The orientation of the triangle shows the increase (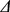) or decrease (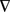) in the accuracy. For example, a white 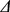 in the naïve group in the silent condition (left column of (i)) indicates that the accuracy in silence was higher than under noise. A red 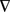 in the conditioned group under the noise condition (right column of (ii)) indicates that, under noise, the accuracy in the conditioned group was lower than that in the naïve group (post-hoc Mann-Whitney U-test after Bonferroni correction for 6 comparisons, p<0.05). (C) Population average of frequency representation accuracies in indicated fields. Asterisks indicate the significance of post-hoc analyses (Mann-Whitney U-test after Bonferroni correction for 6 comparisons: *, p<0.05; **, p<0.01).

Figure 7A summarizes the population averages of frequency representation accuracy in response to 70-dB-SPL tones with varied test frequencies. The accuracies of frequency representations in silence were better than those under the noise condition in 5 out of the 6 frequency ranges in the conditioned group (two-way ANOVA: F(1,2617)  = 24.1–118, p = 9.53E–7–7.36E–27; Post-hoc Mann-Whitney U-test after Bonferroni correction for 6 comparisons: Z = 3.66–8.53, p = 0.00153–8.49E–17), while this was true only in 3 out of 6 test frequencies in the naïve group (Post-hoc test: Z = 2.90–6.01, p = 0.0222–1.09E–8). Although the accuracies were not significantly different between the naïve and conditioned group in silence (Mann-Whitney U-test after Bonferroni correction: Z<2.37, p>0.106), the accuracies were shown to be deteriorated in 4 out of 6 test frequencies under the noise condition after conditioning (two-way ANOVA: F(1,2617)  = 7.42–20.6, p = 0.00649–5.94E–6; Post-hoc test: Z = 3.94–5.03, p = 4.88E–4–2.93E–6); in 5.0, 10 and 40 kHz, the interactions between naïve-conditioned groups and silent-noise conditions were also significant (two-way ANOVA: F(1,2617)  = 4.16–4.81, p = 0.0415–0.0283). Yet, no deterioration of representation accuracy was found in response to the 20-kHz CS tone (two-way ANOVA: F(1,2617)  = 1.22, p = 0.270; Post-hoc test: Z = 1.55, p = 0.733).

Figure 7B further quantifies the accuracies of frequency representations for recording sites with a given BF. Firstly, when the silent and noise conditions were compared, accuracies in silence were better than those in noise in 8 test pairs of tone frequency and BF in the naïve group and 12 in the conditioned group (two-way ANOVA: F(1, 340–577)  = 4.64–182, p = 0.0333–1.02E–34; Post-hoc Mann-Whitney U-test after Bonferroni correction: naïve, Z = 2.75–7.16, p = 0.0360–4.84E–12; conditioned, Z = 2.89–8.44, p = 0.0228–1.86E–16). Secondly, when the naïve and conditioned groups were compared, the conditioning was found to have deteriorated the representation accuracies in 7 test pairs of tone frequency and BF under the noise condition (right column of Fig. 7B (ii)) (two-way ANOVA: F(1, 340–577)  = 4.12–14.9, p = 0.0430–1.25E–4; Post-hoc test: Z = 2.68–4.01, p = 0.0438–3.72E–4), while only 2 significant effects were found under the silent condition (two-way ANOVA: F(1, 571 and 577)  = 9.07 and 5.02, p = 0.00271 and 0.0254; Post-hoc test: Z = 2.72 and 3.10, p = 0.0396 and 0.0114); a significant interaction between the naïve-conditioned groups and silent-noise conditions was found at 40-kHz BF sites in response to 5-kHz tone (two-way ANOVA: F(1,340)  = 10.2, p = 0.00153). Thus, the noise-induced deterioration of frequency representation in the conditioned group was again found only in response to off-CS tones, but not to 20-kHz CS tone at any BF recording sites. On the other hand, noise-induced deterioration at off-CS frequency was mainly found at recording sites with a BF around 20 kHz (i.e., 10, 20 and 40 kHz).

Figure 7C shows the frequency representation accuracy in each field, indicating that the deteriorated accuracies were more frequently found in the core cortex (A1 + AAF) rather than the belt (VAF + SRAF) (two-way ANOVA: for main effect, F(1, 847–939)  = 4.29–18.1, p = 0.0387–2.33E–5 (Post-hoc test: Z = 2.93–3.29, p = 0.0198–0.00602); for interaction term, F(1,939)  = 8.45, p = 0.00373 (Post-hoc test: Z = 3.58, p = 0.00209)).

## Discussion

We performed a context-dependent auditory fear conditioning, in which a mild electrical foot shock as US was associated with 20-kHz CS tone under the noise condition, while only the CS was presented in the silent condition. We demonstrated that this conditioning changed both behavior and auditory cortical activities in a context-dependent manner. After conditioning, although distinct plasticity was not found in the tonotopic map of the auditory cortex (Fig. 4A), tone-evoked responses became more noise-resistive than those pre-conditioning did (Table 1). In CRF, the conditioned group showed reduced spread of neural activation to a given tone in noise, but not in silence. This reduced CRF was associated with sharpening of FRA at 10-kHz BF sites, i.e., just below CS frequency (Fig. 5). The encoding accuracy index of neurons shows that conditioning significantly deteriorated the accuracies of tone-frequency representations in noise at off-CS regions, but not at CS regions (Fig. 7). Because all cortical activities were investigated under anesthesia, the present results support our hypothesis that context-specific learning enables pre-attentive, bottom-up modulation of cortical activities.

### Methodological consideration

Because background noise causes masking effects and alters tone-evoked activities without learning, our experiments were unable to rigorously separate whether context-dependent activities were due to the learning or masking effects. We therefore evaluated our results on the basis of two-way ANOVA, which statistically evaluated the interaction as well as the main effects of learning and context. The significant interactions in behavioral tests indicated that rats were successfully conditioned in a context-dependent form. In terms of neural representation, some significant interactions in the encoding accuracy index indicated that this learning induced context-dependent plasticity in the auditory cortex (Fig. 7). For a number of test parameters in the tuning bandwidth (Fig. 5) and encoding accuracy index (Fig. 7), the context-dependent plasticity was not so clear that, despite significant main effects in both masking and learning, the interaction term in two-way ANOVA did not reach the significant level; yet, post-hoc tests implied that the learning-induced plasticity occurred only under the noise condition.

Based on these analyses, the main finding was the differences between naïve and conditioned groups under the noise condition, but not in silence. The control data in silence, in which there was no sign of learning effect, have guaranteed that the context of noise was needed to reveal the learning effects. This has also excluded the possibility that the learning simply enhanced activities to the CS tone irrespective of the context, making the present study distinct from previous studies [18], [54]–[56]. Thus, our data are sufficient to provide compelling evidence of learning-induced, context-dependent cortical activities.

However, it was impossible to disambiguate whether the learning modified CS-specific tuning properties or masking properties. Both properties were likely modified because the conditioned group was different from the naïve in neural activities under noise to both CS and non-CS tones. To address these questions, additional experiments are required, in which CS tones with different frequencies are used or the US is paired with the CS in silence but not in noise. Alternatively, non-auditory stimulus would be more suitable as contextual information.

### Neural representation in the auditory cortex under noise

The auditory cortex is crucial for signal discrimination in noise, and foreground-background decomposition of sound information, e.g., speech identification in noise [22]–[25], [57], [58]. Continuous background noise reduces activations at the earliest level of auditory system, i.e., cochlear nerve fibers [59], [60]. In the auditory cortex, continuous noise also elevates tone thresholds, and prolongs the latency of tone-evoked activities [61]–[67], which are consistent with our results, i.e., prolonged latency (Fig. 2B) and reduced activation at low intensities (Fig. 5A). Additionally, the encoding accuracy index indicated degraded accuracies of frequency representation in noise (Fig. 7), which are the possible neural correlates of noise-induced masking effects. The prolonged latency in noise suggests that the auditory cortex uses a prolonged time window to obtain sound information from degraded encoding due to background noise [68].

### Learning-induced, context-dependent plasticity in the auditory cortex

Tone-evoked responses became more noise-resistive in the conditioned group than in the naive group (Table 1), providing evidence that learning-induced plasticity in the auditory cortex occurs in a context dependent manner. In addition, the bandwidth decreased in noise, but not in silence (Fig. 5C). These results also implied context-dependent plasticity, because decreases of threshold and bandwidth are typical hallmarks of plasticity in conventional conditioning [42], [69]–[71].

Specifically, we found plasticity in off-CS regions when test tones had a high intensity, which was comparable to the CS-tone intensity. Our experiments were unable to rigorously separate the noise-dependent and CS-specific effects in the conditioning. These synergic effects may result in plasticity in off-CS regions. Alternatively, some plasticity in distant regions of CS frequency, e.g., 5 kHz, should be non-frequency specific, possibly caused by the non-lemniscal auditory pathway [72], [73]. Other off-CS plasticity in the proximity of CS frequency, i.e., 10 kHz and 40 kHz, possibly makes the CS frequency more salient in the cortical representation [5], [74], [75]. Learning-induced, context-specific, plasticity was found as bandwidth sharpening in 10-kHz region (Fig. 5C (ii)) and CF shift in 40-kHz region (Fig. 4B (i)). These plasticity are likely tightly correlated with the learning-induced, context-specific, plasticity of CRF (i.e., the reduced activation shown in Fig. 5B (ii)).

The encoding accuracy index of neurons has verified that the post-conditioning cortical representation of CS becomes more salient in a context dependent manner; conditioning deteriorated representation accuracies at off-CS non-BF frequencies under noise, resulting in relative improvement of the accuracy at CS frequency (Fig. 7). Provided that the accuracy of sensory representation is correlated with perception sensitivity [23], [76], this suggests that an arbitrary tone is more likely to be perceived as CS tone.

Some representation and plasticity were field-specific. In low-to-middle CF regions, background noise disrupted the tonotopic maps in the belt cortex (VAF + SRAF), but not in the core cortex (A1 + AAF), suggesting that the belt cortex accounts for noise-induced deterioration of perception (Fig. 4A (iv)). In terms of learning effects, tone-evoked responses in the belt cortex became more noise-resistive in the conditioned than in the naïve group (Table 1). In addition, learning-induced sharpening of FRA at 10-kHz BF sites occurred only in the belt cortex (Fig. 5E). Conditioning-induced deterioration of the frequency-representation accuracy was less distinct in the belt cortex than in the core (Fig. 7C). These effects in the belt cortex are consistent with a recent study demonstrating that the belt cortex stores long-term emotional memory [20]. Distinctly different anatomical projections also support a functional segregation; the core cortex sends feedback projection to peripheral auditory nuclei but very sparse projections to the limbic and higher cognitive systems, while the belt cortex sends substantial projections to these brain regions [54], [77].

However, we were unable to find distinct CS-specific map plasticity in either the core or belt cortices (e.g., [18], [55], [78]) in a context-dependent form possibly due to the following. Firstly, behavioral salience and motivation (i.e., a stronger shock) may induce more distinct map plasticity [79], but in turn, disrupt context-dependent freezing because of generalization. Secondly, instead of 1-day training in the present study, more lengthy training may induce more distinct plasticity, as the belt cortex is likely to store remote, but not recent, fear memories [20]. Thirdly, because accurate frequency discrimination is not required in our task, the non-lemniscal auditory pathway plays a more predominant role in task execution [73], which induces non-specific plasticity in the auditory cortex [72]. Finally, there is an eventual possibility that cortical map plasticity is not necessary to maintain auditory memory [80], [81].

### Context-dependent cortical modulation of fear memory

Auditory fear conditioning to a single, simple tone induces plasticity in both the lateral amygdala (LA) and auditory cortex [56], [82]. Conditioning-induced increase of responses in the belt cortex is found with longer latencies (20–40 ms) and after more trials (6–9 trials) than in LA (10–20 ms; within 3 trials), suggesting that the direct projection from the auditory thalamus to amygdala is essentially involved in the conditioning. Yet, fear conditioning can be acquired following lesions to the thalamo-amygdala pathways when cortico-amygdala pathways are intact, suggesting that the auditory cortex is also able to directly modulate LA [83], [84]. Some belt neurons show extinction-resistant responses and delayed shock-anticipatory responses [54], [85], suggesting that long-term storage of fear memory and higher cognitive processes in the auditory cortex can modulate amygdaloid activities.

In context-dependent fear extinction, where the hippocampus may play an essential role [31], contextual modulation of tone-evoked responses in LA was found with 40–50-ms post-stimulus latency [26]; in this time range, the conditioning-induced context-dependent cortical responses seen here, as well as other experience-dependent responses, may interact with hippocampal modulations of the amygdala.

Historically, the hippocampus is believed to play a central role in memory retrieval based on contextual cues [28], [86]. Indeed, hippocampal inactivation attenuates context-specific activities in LA, and thereby context-specific fear responses [31]. However, hippocampus-independent learning, i.e., elemental simple cue learning, may be partially effective to encode contextual cues, specifically when such cues are non-spatial [33], [87]. Additionally, neurons in the auditory cortex take part in varied auditory tasks more flexibly than hippocampal neurons [88], while multi-sensory contextual cues modulate hippocampal neurons more dynamically than auditory cortical neurons [89]. The hippocampal modulation is likely independent of the auditory cortical modulation, because the connection between the auditory cortex and hippocampus is indirect; the hippocampus receives auditory input from perirhinal cortex via lateral entorhinal cortex [90] and medial prefrontal areas via the thalamus [91]. Rather, direct projections from the auditory cortex to amygdala support the possibility that the auditory cortex directly modulates amygdala activities according to auditory contexts [54].

In state-dependent learning, memory retrieval is enhanced when the endogenous state and sensory context during encoding are reinstated at the time of retrieval [2]. One of the determinants of endogenous state is cortical levels of acetylcholine, which switches receptive fields by alternating recurrent inhibitory pathways [92], and increasing thalamo-cortical transmission [93]. Other neuromodulators such as noradrenaline and dopamine also modulate receptive fields and gating of information [94]–[97]. Such an endogenous state is the subject of attentive control [98]. In contrast, the ongoing sensory context pre-attentively modulates neural activities through rapid synaptic depression [8], [15] and other intrinsic network properties [11], [16]. Such modulation may enable state-dependent computations [99]. The present study indicates further investigations to elucidate how the auditory cortex recognizes the ongoing state, and associates a specific tone with fear memory in a context-dependent manner.
